# Magnetic microparticle concentration and collection using a mechatronic magnetic ratcheting system

**DOI:** 10.1371/journal.pone.0246124

**Published:** 2021-02-18

**Authors:** Oladunni B. Adeyiga, Coleman Murray, Hector E. Muñoz, Alberto Escobar, Dino Di Carlo

**Affiliations:** 1 Division of Infectious Diseases, David Geffen School of Medicine, Department of Medicine, University of California, Los Angeles, Los Angeles, California, United States of America; 2 Ferrologix, Inc., Redondo Beach, California, United States of America; 3 California NanoSystems Institute, Los Angeles, California, United States of America; 4 Department of Bioengineering, University of California, Los Angeles, Los Angeles, California, United States of America; 5 Department of Biomedical Engineering, University of California, Irvine, Irvine, California, United States of America; The Ohio State University, UNITED STATES

## Abstract

Magnetic ratcheting cytometry is a promising approach to separate magnetically-labeled cells and magnetic particles based on the quantity of magnetic material. We have previously reported on the ability of this technique to separate magnetically-labeled cells. Here, with a new chip design, containing high aspect ratio permalloy micropillar arrays, we demonstrate the ability of this technique to rapidly concentrate and collect superparamagnetic iron oxide particles. The platform consists of a mechatronic wheel used to generate and control a cycling external magnetic field that impinges on a “ratcheting chip.” The ratcheting chip is created by electroplating a 2D array of high aspect ratio permalloy micropillars onto a glass slide, which is embedded in a thin polymer layer to create a planar surface above the micropillars. By varying magnetic field frequency and direction through wheel rotation rate and angle, we direct particle movement on chip. We explore the operating conditions for this system, identifying the effects of varying ratcheting frequency, along with time, on the dynamics and resulting concentration of these magnetic particles. We also demonstrate the ability of the system to rapidly direct the movement of superparamagnetic iron oxide particles of varying sizes. Using this technique, 2.8 μm, 500 nm, and 100 nm diameter superparamagnetic iron oxide particles, suspended within an aqueous fluid, were concentrated. We further define the ability of the system to concentrate 2.8 μm superparamagnetic iron oxide particles, present in a liquid suspension, into a small chip surface area footprint, achieving a 100-fold surface area concentration, and achieving a concentration factor greater than 200%. The achieved concentration factor of greater than 200% could be greatly increased by reducing the amount of liquid extracted at the chip outlet, which would increase the ability of achieving highly sensitive downstream analytical techniques. Magnetic ratcheting-based enrichment may be useful in isolating and concentrating subsets of magnetically-labeled cells for diagnostic automation.

## Introduction

As early as 1792, the use of magnetism and magnetic materials in separation applications has been documented [1, 2]. Introduction of the magnetic-activated cell separation (MACS) systems, which couple small superparamagnetic particles bound to cells with high gradient magnetic fields (generated using a ~ 0.6 Tesla external magnet and steel wool columns) to isolate cells, brought a substantial increase in throughput for cell enrichment [3]. With MACS, magnetic based techniques for cell separation and enrichment became a bench laboratory standard. Compared with fluorescence activated cell sorting (FACS) alone, combining MACS with FACS offered the ability to sort 10 million cells in 1 hour, an improvement over a 10 hour FACS process [3]. Since this first report of the use of MACS with FACS to increase cell sorting throughput, additional work has demonstrated the ability separation applications using magnetism to sort 10 million cells per second and to collect human circulating tumor cells, which, when present, are usually in rare amounts within whole blood [4–6]. However, opportunities for improvement in the ability of magnetism based separation applications to isolate and concentrate cells present in liquid suspension have remained. For example, researchers have continued to need to rapidly isolate small populations of specifically immunolabelled cells present within a large background of unlabeled cells in an automated fashion, a strength of FACS. There also exists the need to more discriminately separate cells based on the quantitative amount of magnetic labelling present on that cell. While MACS has been applied to this purpose [7], its limited widespread use for these functions has motivated continued research to broaden the functionality of magnetism for highly resolved cell separation and enrichment. Since then, magnetic separation approaches have been incorporated into microfluidic devices for cell and particle separation applications, giving rise to the field of micro-magnetofluidics (MMF) [8–16]. MMF approaches take advantage of laminar microscale device flow physics [17], allowing highly resolved, controlled, magnetic-based separation.

Using MMF techniques, flow-based and ratcheting-based magnetic separation can be performed. Flow-based separation exploits physical properties or flow characteristics of the fluid flow in order to separate magnetic entities. Laminar fluid flow can be used to focus and align cells, followed by deflecting cells with magnetic force in a controlled manner [11, 12, 18, 19]. Contrast this with ratcheting-based magnetic separation, in which case rectified magnetic particle movement, where the magnetic force acting on the particle causes deterministic particle movement, is achieved by modifying the magnetic potential energy landscape of a surface magnetized in the presence of an external magnetic field [20]. In contrast to flow-based separation, magnetic ratcheting offers the ability to achieve highly resolved and controlled magnetic particle movement, based on magnetic content, for on-chip applications [20, 21]. Magnetic ratcheting brings the possibility of achieving a level of controlled cell separation and concentration to discrete locations on a substrate, a substantive advance for lab-on-chip devices [21, 22].

Using magnetic ratcheting, we previously reported the use of a chip design consisting of magnetic elements placed with a gradient in pitch to realize a quantitative magnetic separation technology [23–26]. In these previous reports, the chip used contains rows of magnetically soft (i.e. with low magnetic hysteresis), high aspect ratio magnetizeable micropillars that are arranged with increasing distance between pillars, i.e. increasing horizontal pitch *(P)*. A high aspect ratio magnetizeable micropillar has an increased magnetic force capacity, so that smaller superparamagnetic particles with increased binding efficiency for cell immunolabeling can be used. In contrast to the magnetic force present in bulk magnetic field extraction techniques, the magnetic force exerted locally on magnetic particles is greatly increased with this design, improving magnetic ratcheting robustness. Using a varying pitch, increasing horizontal pitch allows quantized concentrated particle band separation according to magnetic content; magnetic particles with higher iron oxide content will concentrate at locations where the micropillar array has larger pitch. Magnetic particles (free or cell-bound) will then localize to separate chip regions based on quantitative magnetic content.

**Fig 1:**
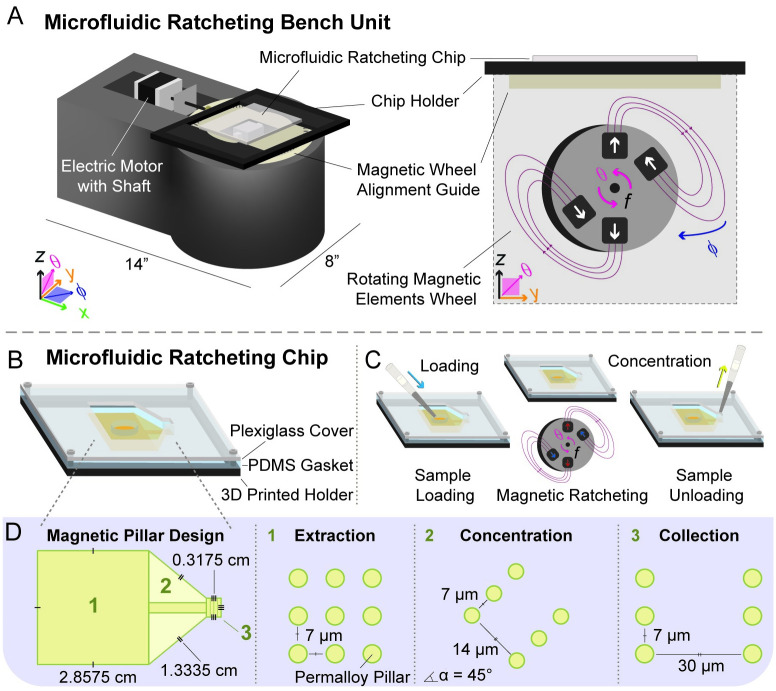
Microfluidic magnetic ratcheting unit and chip. A) An isometric drawing of the unit, which consists of a 3D printed unit base, a motorized unit, a rotating wheel of N52 grade rare Earth magnets arranged in a partial Halbach array, and enclosure which contains the microfluidic ratcheting chip. An enlarged illustration of the partial Halbach magnet array demonstrates the magnetic field generated by the wheel over multiple wheel rotation cycles. The wheel rotates in the y-z plane with frequency (*f*) that is established by the rotational velocity (*ω*), where *ω = 2πf = dθ/dt*. B) The microfluidic ratcheting chip is within an enclosure that also contains a Plexiglass cover, PDMS gasket, and 3D printed holder. C) Fluid processed on the chip surface is introduced into the chip inlet region and removed from the chip outlet region using a micropipetter. D) This chip design contains three regions, with different permalloy pillar array geometries.

Along with separation, magnetic ratcheting can be used to concentrate magnetically tagged entities, such as mammalian or microbial cells bound to magnetic particles, to enable detection with an increased level of sensitivity, which is the focus of this work. We use a cost-effective mechatronic system (Fig 1), a unit containing both a rotating wheel containing a partial Halbach array of N52 grade rare Earth magnets (dimensions 2.5 cm x 2.5 cm x 1 cm, with a surface magnetic flux centered on the axis of magnetization of 0.4933 T) and an enclosure within which the newly designed magnetic ratcheting chip is placed. The unit for this experimental work can be described as follows: using the Cartesian coordinate system as a point of reference, the rotating wheel lies within the y-z plane, and the enclosed magnetic ratcheting chip lies within the x-y plane. The rotating wheel is located beneath the enclosed magnetic ratcheting chip, and with its rotation axis orthogonal to the normal vector of the plane of the enclosed magnetic ratcheting chip. Two rotational angles give the position of the rotating wheel with respect to the enclosed magnetic ratcheting chip. Within the x-y plane, the azimuth angle phi *(φ)* can be defined as the angle between the rotating wheel outward surface normal vector and the magnetic ratcheting chip long axis. Before each experimental run, this angle is fixed. Within the y-z plane, the polar angle theta *(θ)* can be defined as the angle between a radial vector on the magnetic wheel and the magnetic ratcheting chip long axis. During each experimental run, the wheel rotates with frequency (*f*) that is established by the rotational velocity (*ω*), where *ω = 2πf = dθ/dt*.

**Fig 2:**
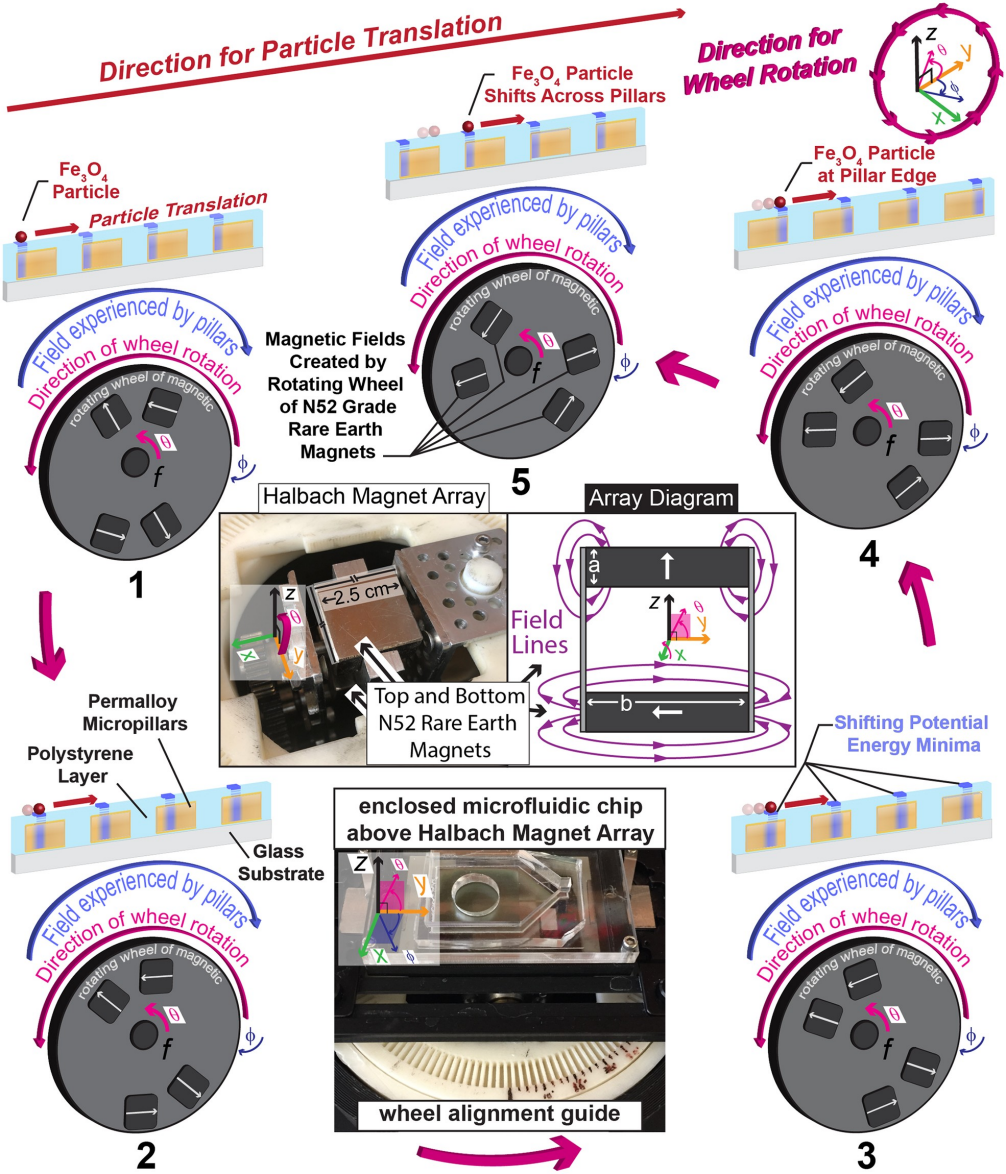
Surface particle movement dynamics. Illustrated magnetic particle movement dynamics; as the mechatronic wheel cycles, MPs translate across chip surface. The rotating wheel, which contains two N52 grade rare Earth magnets (2.5 cm x 2.5 cm x 1 cm) connected with two nonmagnetic metal bars, rotates around the x axis and generates surface magnetic potential energy wells. The width, *b* = 2.5 cm is shown as well as the depth, *a* = 1 cm. Shifting surface magnetic potential energy minima locations make rectified magnetic particle movement energetically favorable. Here, particle movement over the chip surface which has been planarized by a polystyrene layer is displayed in five stages, where, between stage 1 and stage 5, a particle is translated from one pillar to the next pillar, but not to the preceding pillar because this is energetically unfavorable. An illustration of the magnetic field generated over multiple wheel rotations is shown, along with a photo of the partial Halbach magnet array.

**Fig 3:**
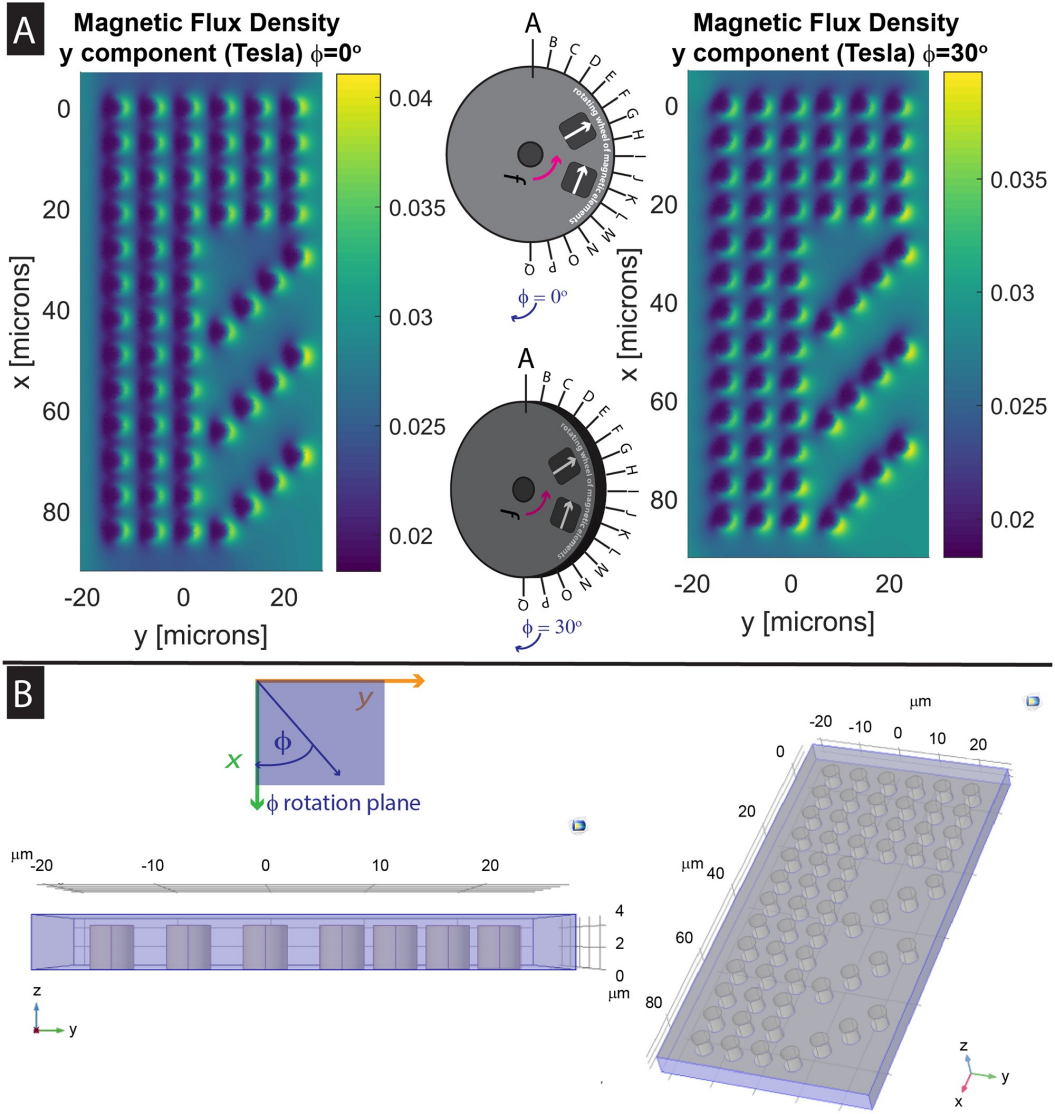
Simulation data plots. A) Magnetic flux density contours from COMSOL^®^ (COMSOL, Inc.) simulation data which has been displayed using MATLAB^®^. Demonstrated magnetic contours display chip surface magnetic flux density with the wheel position at position A, angle phi *(ϕ)* = 0° & 30°. High gradients in flux density correspond to regions where magnetic particles are phase locked as the external field cycles. In comparing displays for the two wheel positions, there is a difference in the angle of the field minima, in comparison to the magnetic pillar structures. These pillar structures represent the region of the chip where region 1 and region 2 meet. B) COMSOL Model. Using COMSOL, a 3D model displaying the geometry of the magnetic ratcheting chip was created. The displayed pillar structures represent the region of the chip where region 1 and region 2 meet. The chip contains permalloy pillars with 4 μm diameter, which are covered by a 1 μm thick polystyrene layer.

In contrast to the previously reported work, here we present a new design for a magnetic ratcheting chip that has three distinct regions (Fig 1). In region 1, micropillars are patterned in an array with equal spacing along the x and y axis and with a single pitch. In region 2, which is immediately adjacent to region 1, micropillars are patterned in rows at a 45 angle to the y axis, also with a single pitch. Finally, in region 3, micropillars are patterned in rows along the x axis, with a single pitch, in order to act as a collecting region. The surface of the chip has been planarized by coating the magnetic ratcheting chip with a thin layer of polystyrene (Figs 2 and 3). The rotating wheel applies externally controlled magnetic fluxes in a cyclic manner to the chip (Fig 2). Through a series of batched fluid experiments, we 1) visualize the dynamics of on chip magnetic particle movement, 2) determine final magnetic particle location following ratcheting, based on wheel orientation, 3) empirically determine the optimal wheel orientation to allow magnetic particles to be ratcheted to a localized point on the chip, 4) quantify the chip concentrating ability, and 5) demonstrate the system’s ability to concentrate differently sized magnetic particles. We believe this is a potentially useful technique to enrich magnetically-labeled cells, including bacteria, in diagnostic and therapeutic applications.

## Results

### Directed magnetic ratcheting using a mechatronic system

Using this mechatronic unit (Fig 1), magnetic particles introduced to the chip loading region are directed towards the concentration region over time. Directed particle motion on-chip requires the presence of the rotating mechatronic wheel of rare Earth magnets, in order to amplify the magnetic field gradients at the local scale. In contrast, a static magnetic field does not induce magnetic particle movement when used with the magnetic ratcheting chip. Using a stationary N52 grade rare Earth magnet with dimensions 2.5 cm x 2.5 cm x 1 cm, a surface magnetic flux centered on the axis of magnetization of 0.4933 T, and a pull force that rapidly decays with distance from the magnet, magnetic particles remain stationary in the control experimental set up (S1 Fig). We used iron oxide composite particles with diameter of 2.8 microns (magnetic susceptibility = 6.10 x 10^−4^ m^3^ · kg^-1^, Fe content = 14%, density = 1.6 g · cm^-3^, magnetophoretic mobility not reported) (ThermoFisherScientific, Technical Data)), diameter of 500 nm (*H* = 80 kA/m, density = 3.0 g · cm^-3^, magnetophoretic mobility not reported) (Micromod, Technical Data Sheet), and diameter of 100 nm (*H* = 80 kA/m, density = 3.2 g · cm^-3^, magnetophoretic mobility not reported) (Micromod, Technical Data Sheet). In our experimental set up, the stationary magnet is 3 cm from the edge of the magnetic ratcheting chip. At this location, the measured pull force for the magnet alone is small (K&J Magnetics, Inc., Technical Data).

In the simulation work, where we plot magnetic flux density for the surface of the magnetic ratcheting chip, when the chip is in proximity to the rotating wheel, the plotted magnetic flux density (Fig 3) shows a large gradient near the magnetic pillars. This results in amplified local forces that can trap magnetic particles locally, but with trapping locations that ratchet as the field direction changes with the magnetic wheel rotating in the y-z plane, in positions defined by polar angle theta *(θ)* (Fig 3). The plotted magnetic flux density on the chip surface that does not contain magnetic pillars, however, is much smaller. This demonstrates how the local fields induced by the magnetized permalloy elements remain local in the region surrounding each pillar. In addition, with simulation work, there is a demonstrated shift in the magnetic ratcheting chip surface magnetic field contours present around each pillar that occurs when the azimuth angle for the system is shifted, which was further investigated experimentally. For initial conditions, wheel surface magnet field measurements were performed at 11.25° intervals, wheel positions A–Q, were taken (Fig 3) [27].

### Effect of partial Halbach magnet array wheel azimuth angle phi (φ) on magnetic ratcheting concentration

With numerical simulations, performed using COMSOL^®^ (COMSOL, Inc.) for model creation and simulations, the chip surface magnetic flux density for each pillar could be determined (Fig 3). A preceding study of 2.8 μm magnetic particles demonstrated the dynamic potential wells present on each micropillar surface, considering pillar positioning present in chip region 1, using data from COMSOL simulations to calculate the chip surface force density at set time points through the ratcheting cycle [27]. In this work, we extended the simulations to calculate chip surface magnetic flux density near each micropillar surface, considering pillar positioning present in chip region 2. In region 2, micropillars are patterned in rows at a 45 angle to the y axis, whereas in region 1, micropillars are patterned in rows along the y axis, or long axis of the magnetic ratcheting chip. From the output of these simulations, there is a demonstrated difference in magnetic contours. This shift in magnetic contours gives some insight regarding how shifting the azimuth angle phi impacts surface magnetic particle movement, which required further investigation.

Experimentally, we investigated what was the optimal azimuth angle phi *(φ)* for the rotating magnetic wheel to obtain a single point of concentration for 2.8 μm superparamagnetic iron oxide particles. Magnetic particles were observed to concentrate at three chip surface locations with wheel orientation at angle phi *(φ)* = 0° (S2 Fig). To drive particles into one location for concentration and collection, additional system orientations were evaluated. A wheel orientation at angle phi *(φ)* = 30° biases on-chip particle movement such that the majority of particles concentrate at one location (S3 Fig). Examining the shifting location of chip surface magnetic flux density gradients could explain this behavior (Fig 3). Given the highest concentration at this angle, further experiments varying particle size, duration and frequency were performed using a wheel orientation at angle phi *(φ)* = 30°.

### Time and frequency of magnetic ratcheting

We studied the effect of varying ratcheting frequency and duration in this system on the resulting time-dependent magnetic particle concentration for 2.8 μm superparamagnetic iron oxide particles present within an aqueous suspension. To determine this, a concentration factor (CnFc) was calculated:

$$CnFc=\frac{{\;[Beads]}_{outlet}}{{\left[Beads\right]}_{inlet}}\;∙100\%$$ (1)

**Fig 4:**
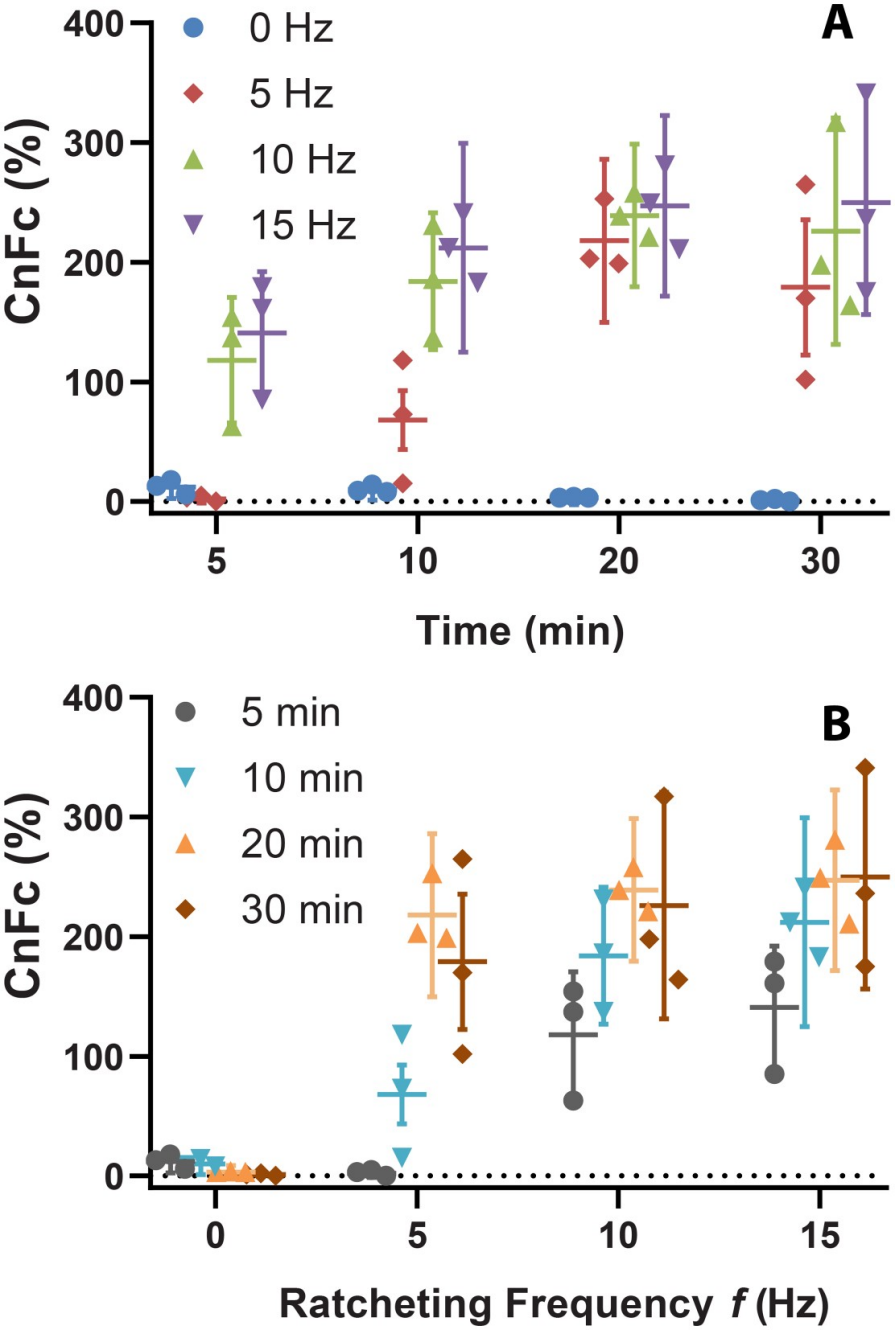
Concentration factors for experiments with 2.8 μm particles. Using azimuth angle phi *(φ)* = 30°, CnFc (calculated using Eq 1) is shown to increase with A) ratcheting time and B) frequency for 2.8 μm particles. Mean CnFc for *N* = 3 trials with standard error calculation is displayed. Experimental data are displayed in two ways to illustrate how changing frequency and time act in similar ways to influence system output, emphasizing that time and frequency are two parameters that control concentration factor. For these experiments, particle suspensions were created by adding 10 μL of bead stock (6-7E8 beads per milliliter) to 990 μL of a 1% (w/v) BSA-PBS liquid mixture, and a total of 1 mL was introduced onto the chip.

As ratcheting time increases, the magnetic particle ratcheted amount increases (Fig 4), reaching a maximum after 20 minutes for each tested frequency, and saturating as all loaded particles accumulate at the concentration region. Increased frequency can also reduce needed ratcheting time, where increased frequency leads to more rapid bulk magnetic particle motion. With increases in both ratcheting time and frequency, a further increase in CnFc was noted. We tested several conditions, including ratcheting frequencies of 0, 5, 10, and 15 Hz; also, a maximal time of 30 minutes. Comparing CnFc results at 20 and 30 minutes, a minimal increase in CnFc occurs, indicating a saturation point where most particles are concentrated after 20 minutes. A CnFc of greater than 200% was ultimately achieved following ~10 minutes of operation for the highest frequencies. Particles suspended in a ~1 ml volume of fluid, covering a chip surface area of 992 mm^2^, are finally concentrated into a small surface area footprint (~10 mm^2^), a ~100-fold surface area concentration, which could be helpful for more precise optical imaging or sensing in a small field of view. Higher CnFc could be achieved by removing a smaller outlet volume from this concentration patch than the ~300 μL currently extracted.

### Effect of particle size on magnetic ratcheting dynamics

**Fig 5:**
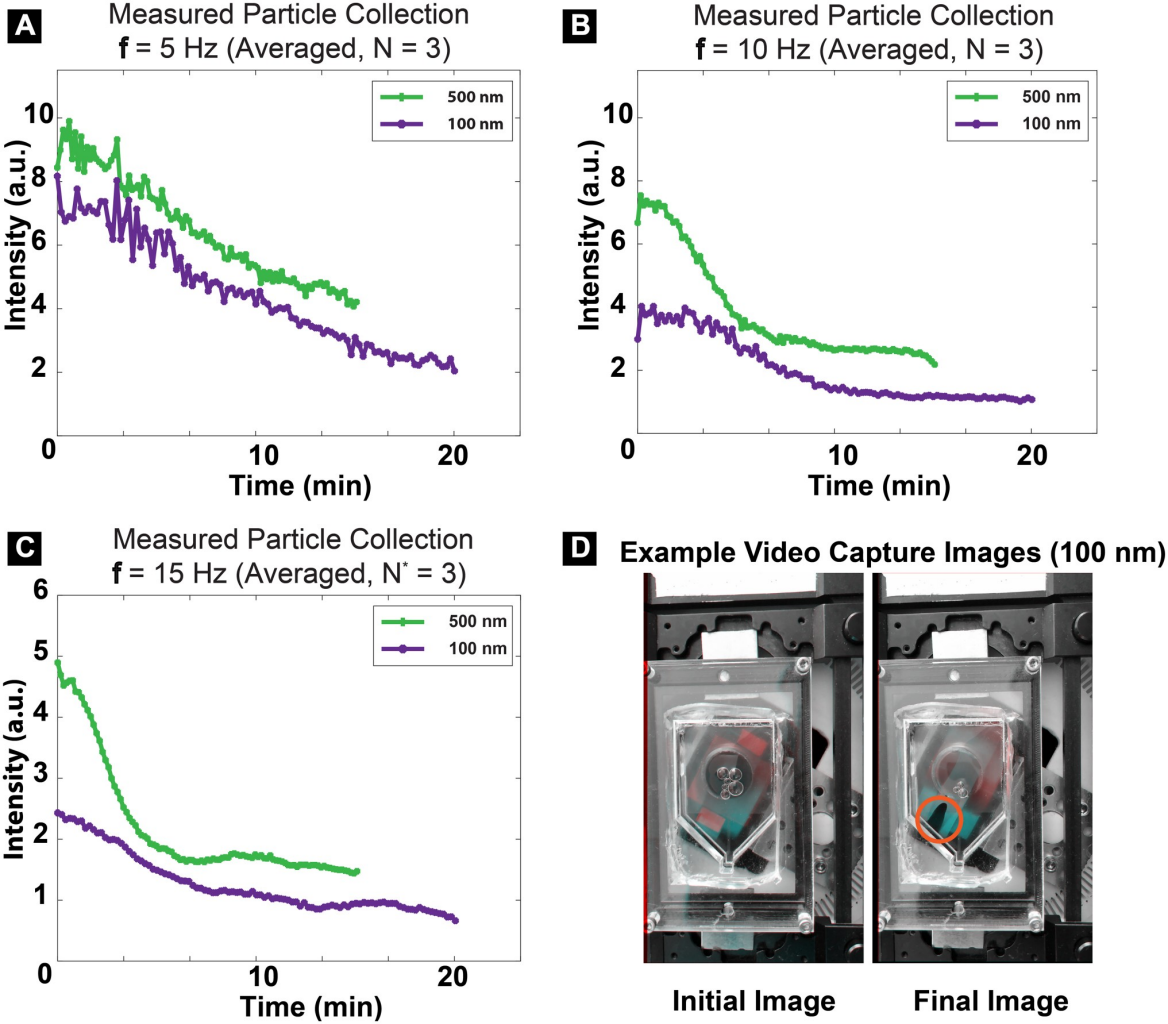
Ratcheting experiments with 500 nm & 100 nm magnetic particles. A) Ratcheting experiments demonstrating ratcheting time needed for particle accumulation in the collection region. A-C) 100 nm and 500 nm particles accumulate, with 100 nm particles needing a longer ratcheting time. Intensity represents the light intensity in the collection region which decreases as particles accumulate and transmitted light is blocked. D) Example images from time lapse video recordings demonstrate how collected particles accumulate within the collection region. For these experiments, particle suspensions were prepared by diluting particle stock into PBS, obtaining concentrations of ~5.1E7/mL for 500 nm and ~3.0E9/mL for 100 nm magnetic particles.

We have further demonstrated the ability of the system to ratchet and concentrate smaller magnetic particles (100 nm and 500 nm). This is notable given the dependence of magnetic force on magnetic volume of the particle. We used the same magnetic volume for each experimental condition (1.43E8 cubic micrometer · millileter^-1^). Magnetic particle concentration, as demonstrated by a visible concentrated pack of particles, occurs for 100 nm particles after ~16 minutes, for 500 nm particles after ~12 minutes, and for 2.8 μm particles after ~10 minutes (Fig 5, S4–S6 Figs). The time needed for maximal particle collection is determined by measuring the time needed for light intensity in the particle collection region to decrease, which corresponds with particle collection. We see that smaller particles require more time for collection to occur, perhaps due to particle-particle interactions and crowding during ratcheting. However, given the strong amplification of the magnetic field gradient, we demonstrate that we are able to collect particles of varying sizes, using this technique. Each batch experiment was performed using an aqueous liquid suspension containing a monodisperse, homogeneous suspension of particles.

## Discussion

Regarding the surface transport and concentration of magnetic particles in this system, the physical phenomenon known as magnetic ratcheting is used. In considering an individual magnetic particle existing on the surface of the magnetic ratcheting chip, which has been exposed to an external magnetic field, the particle will move, in response to the local gradient now present within regions near each permalloy micropillar, and reach a local equilibrium position. For effective ratcheting, the timescale for reaching a local equilibrium is small, as compared with the exchange or transition rate between each state. Here, the main state change is the movement of magnetic particles between neighboring local equilibrium positions, which can span between two neighboring pillars. The concentration effect, in which particles dispersed in a liquid suspension become aggregated into one location on the chip surface, is achieved if magnetic particles are directed to a converging location on the chip. This occurs by shifting the azimuth angle (*ϕ*), where the optimal angle between the radius of the rotating wheel and the long axis of the chip, has been determined.

The magnetic force ***F***_*m*_ on magnetic particles is given here:

$$F_{m}=(m\nabla )B=\mu_{o}(m\nabla )H=\frac{1}{2}\;\mu_{o}\chi_{MP}V_{MP}\nabla H^{2}$$ (2)

Here, magnetic particle physical properties include ***m***, magnetic moment, equal to *χ*_*MP*_*V*_*MP*_***H***,; *χ*_*MP*_, magnetic susceptibility, *V*_*MP*_, MP volume; and magnetic field strength ***H***, defined as ***B****/μ*_*o*_. Integrating Eq (2) gives MP potential energy ***U***_*m*_:

$$U_{m}=-\int F_{m}=-\frac{1}{2}\mu_{o}\chi_{MP}V_{MP}H^{2}$$ (3)

These equations apply given that our system works within a regime where magnetic field strength is low, magnetic particle magnetization is not saturated, and magnetic susceptibility is constant. Furthermore, within magnetic particles, ***H*** is curl-free; the (***H***∇)***H*** term from Eq (2) can be rewritten as $\frac{1}{2}\;\nabla H^{2}$ [28]. For higher strength fields, achieved with stronger permanent magnets, the relationship between magnetic particle magnetization and the applied magnetic field is not linear, so *χ*_*MP*_ is no longer constant. The magnetic particle suspension media is primarily water; its magnetic susceptibility is therefore neglected in these equations [29]. The equations given here along with fluid dynamic drag yield a set of equations governing the ratcheting behavior.

Our results indicate smaller particles require increased ratcheting time for collection which agrees with theoretical predictions. The following equations govern chip surface particle transport [23, 27]:

$$\bar{F_{mag}}=\frac{V_{p}\chi_{MP}}{\mu_{o}}\bar{(B\cdot \nabla )B}$$ (4)

$$\bar{F_{mag_{y}}}=\frac{V_{p}\chi_{MP}}{\mu_{o}}F_{den}\;\text{sin}(\frac{2x_{p}}{P}-2\omega_{ext}t)$$ (5)

$$\bar{F_{drag_{y}}}=6\pi \mu_{f}r_{p}\bar{u_{p}}$$ (6)

$$\bar{u_{p_{y}}}=\frac{1}{6\pi \mu_{f}r_{p}}\frac{V_{p}\chi_{MP}}{\mu_{o}}F_{den}\text{sin}(\frac{2x_{p}}{P}-2\omega_{ext}t)$$ (7)

$\overset{-}{F_{mag}}$ is time averaged magnetic force, $\overset{-}{F_{drag}}$ is time averaged drag force, *χ*_*MP*_ is particle susceptibility, *μ*_*o*_ is free space permeability, ***B*** is magnetic flux density, *F*_*den*_ is average force density, ***x***_*p*_ is particle position, *P* is pitch, *ω*_*ext*_ is ratcheting frequency, *t* is time, *μ*_*f*_ is fluid viscosity, *r*_*p*_ is particle radius, *V*_*p*_ is particle volume, and $\overset{-}{u_{p}}$ is particle velocity. In considering magnetic particle transport along the chip surface, magnetic particle movement is characterized by balancing time-averaged magnetic and drag forces [23, 27]. Increasing rotating wheel frequency increases the time-averaged magnetic force and velocity experienced by magnetic particles, until the frequency exceeds a critical value where particles do not have sufficient time over a cycle to reach the next pillar. Our experiments reflect that this critical value is not exceeded as we find that concentration time decreases monotonically with increasing ratcheting frequency across particles of different sizes. For example, ratcheting time and frequency were in a trade-off relationship (Fig 4). Our concentration time also appears to relatively independent of particle size, which indicates that particles of all sizes were entrained across the ratcheting frequencies tested.

## Conclusion

In conclusion, we have shown the ability of magnetic ratcheting on a flat microchip substrate for the purpose of concentrating superparamagnetic particles of varying sizes. With this new chip design, magnetic ratcheting cytometry can be further developed to allow the concentration of mammalian cells, bacteria, or other analytes of interest tagged with magnetic particles, with potential as a pre-treatment approach for medical diagnostics to purify and concentrate diagnostic cells associated with a disease. Plans for future work include investigation using simulation methods to further understand how shifting the azimuth angle (*ϕ*) determines final particle location, determining the limits of sensitivity for small amounts of magnetic particles would be useful to explore in future studies, and demonstrating the ability of this system to be used for concentrating bacteria in various clinical specimens.

## Materials and methods

Chips were fabricated using a previously reported technique [23]. Briefly, chips containing nickel-iron permalloy cylindrical micropillars, with ~4 μm thickness and 1:1 aspect ratio, were fabricated in a cleanroom by electroplating onto a SPR220 resist mold patterned on a Ti-Cu-Ti seed layer coated onto borosilicate glass slides via e-beam evaporation, with intervening etching and stripping steps. For smooth magnetic particle movement, chip surface was planarized with a ~1 μm thick polystyrene layer, a procedure repeated after three uses. Following stripping, rinsing, and drying steps, chips were placed in a hexamethyldisilazane (HMDS) chamber, then had a 5% (w/v) polystyrene-toluene mixture spun on. Before use, chips were soaked in a 2% (w/v) pluronic-deionized water mixture, then washed and air dried. The chip has three regions; Region 1, where magnetic particles are extracted from the loaded bulk fluid, Region 2, where magnetic particles are concentrated into a smaller area, and Region 3, where magnetic particles can be collected (Fig 1).

The platform (Fig 1) is a microfluidic magnetic ratcheting chip benchtop unit built for high-throughput enrichment of magnetically-tagged entities present in liquid suspension [30]. Within the unit, an external magnetic field is created with a rotating wheel of a partial Halbach array of N52 grade rare Earth magnets. An enlarged illustration of the partial Halbach magnet array demonstrates the magnetic field generated by the wheel over multiple wheel rotation cycles (Figs 1 and 2). At a radial distance of 3mm from the wheel center, the peak magnetic flux density is 300mT and the average flux density ~ 100mT [27]. The external magnetic field acts on the chip surface to create dynamic potential energy wells leading to the unidirectional ratcheting movement of magnetically-tagged entities (Fig 2). Magnetic particles extracted from the bulk fluid move in the direction opposite to wheel rotation. Particles enriched into a smaller chip surface area can be pipetted off the chip. Variables tested include wheel rotation frequency and time.

To further understand chip surface dynamics, we performed numerical simulations to calculate the generated chip surface magnetic flux density in the presence of the external field created by the rotating wheel. Simulations were performed using the Magnetic Fields, No Currents Stationary Physics Interface in COMSOL Multiphysics^®^ version 5.3a (COMSOL, Inc.), with wheel orientation in the x-y plane at angle phi *(φ)* = 0° and 30° (Fig 3). A model using a geometry containing 4 μm diameter permalloy pillars with relative magnetic permeability of 8500 encased within a 5 μm high rectangular slab with relative permeability of 1 was created (Fig 3). For initial conditions, previously reported wheel surface magnet field measurements at 11.25° intervals were used (Fig 3, at wheel positions A-Q) [27]. Results are taken from simulation data calculated at the top of the rectangular slab (1 μm above the permalloy pillars within the slab), representing the chip surface.

For set up, a watertight seal assembly was formed with a glass slide base, the chip, a polydimethylsiloxane (PDMS) gasket with punched out regions for introducing (fluid inlet surface area region = 9.92 cm^2^) and removing fluid (fluid outlet surface area region = 0.10 cm^2^), and a plexiglass cover (Fig 1). The gasket (impression thickness = 0.013 inch) was created using a laser cut mold in acrylic, and PDMS was cured after a conventional degassing procedure performed on PDMS mixed at a 1:15 crosslinker:base ratio. Chips were primed using a 1% (w/v) Bovine Serum Albumin (BSA)-phosphate buffered saline (PBS) liquid mixture; priming liquid was introduced into the inlet, followed by excess fluid removal, and test liquid particle suspension loading into the loading region using a micropipetter for each experimental run. The assembly was then placed on the 3D printed constructed unit base (Fig 1). Within the unit, the wheel rotates in the y-z plane, with radial position defined by polar angle theta *(θ)*; wheel orientation is defined by angle azimuth angle phi *(φ)* in the x-y plane with respect to the chip (Fig 1). As angle theta *(θ)* changes, the generated cycling external field impinges on the chip surface, including the permalloy structures and magnetic particles. Shifting the angle phi *(φ)* has important implications for final particle destination on chip. Several configuration options set instrument operation mode use parameters, including wheel orientation, time, and frequency for ratcheting. Wheel orientation (operation mode) is set by shifting the magnetic wheel alignment guide (Fig 1) using a custom LabVIEW^™^ (National Instruments) program. After determining the optimal system use settings, we tested the effect of variables such as ratcheting time and frequency.

Experiments were performed using superparamagnetic beads of varying sizes. 2.8 μm beads (Dynabeads^®^ M-280 Streptavidin, ThermoFisher Scientific). Suspensions were created by adding 10 μL of bead stock (6-7E8 beads per milliliter) to 990 μL of a 1% (w/v) BSA-PBS liquid mixture. A total of 1 mL was introduced onto the chip using a micropipetter to introduce fluid, and then was batched processed on chip, with serial image capture used to document magnetic particle movement. For the experiments using 2.8 μm beads, bead suspension concentration before and after ratcheting was determined by counting beads in suspension using a hemocytometer; 294 μL was removed using a micropipetter from the chip concentration region for counting. To determine the ability of this system to ratchet superparamagnetic particles of varying size, we also conducted experiments with 500 nm and 100 nm particles. 500 nm and 100 nm superparamagnetic particle suspensions (Micromod) were prepared by diluting particle stock into PBS, obtaining concentrations of ~5.1E7/mL for 500 nm and ~3.0E9/mL for 100 nm magnetic particles. Suspensions were prepared so as to match magnetic particle volume fraction for each particle size (1.43E8 cubic micrometer · millileter^-1^). Directed particle movement was documented with time lapse video capture over 20 minutes for 100 nm particles, 15 minutes for 500 nm particles, and 10 minutes for 2.8 μm particles, using a ratcheting frequency of 5 Hz. For the experiments performed with 500 nm and 100 nm particles, in order to determine the relative change in the size of particle pack collected, as particles accumulate at one chip location over time, a video analysis of each time lapse video was performed with MATLAB^®^ assistance. This was done by modifying an algorithm written for video stabilization, followed by an automated comparison of the each sequential image with the final image containing the collected particle pack, and calculation of the light intensity for each image [31].

## Supporting information

S1 Fig Stationary magnet control experiment. (DOCX)

S2 Fig Baseline ratcheting experiments. (DOCX)

S3 Fig Ratcheting experiments with adjusted azimuth angle phi (*ϕ*). (DOCX)

S4 Fig Ratcheting experiments at frequency (*f*) = 5Hz. (DOCX)

S5 Fig Ratcheting experiments at frequency (*f*) = 10Hz. (DOCX)

S6 Fig Ratcheting experiments at frequency (*f*) = 15Hz. (DOCX)
