# Supplementary material for: Magnetic microparticle concentration and collection using a mechatronic magnetic ratcheting system
Source: PLoS One. 2021 Feb 18;16(2):e0246124. doi: 10.1371/journal.pone.0246124 (PMC7891735; doi:10.1371/journal.pone.0246124)
Supplement: S1 Fig — (DOCX) [file pone.0246124.s001.docx]

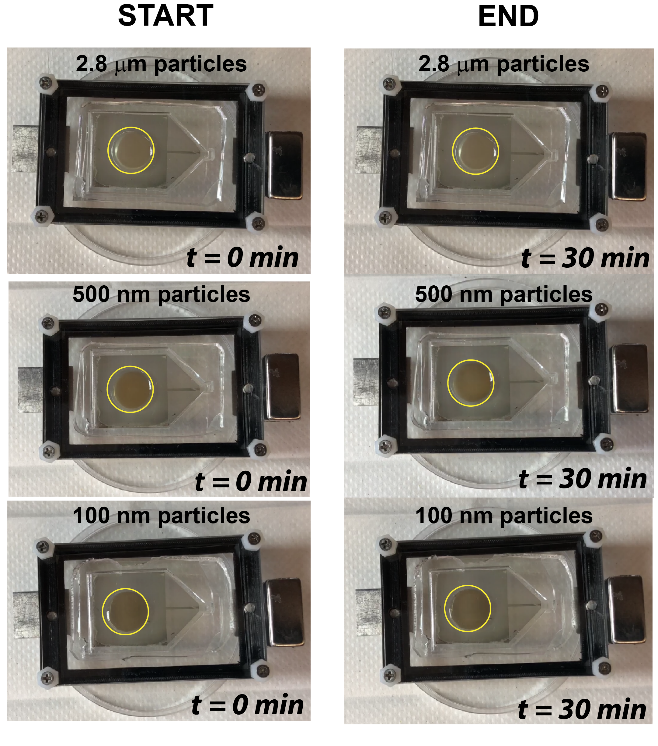


S1 Fig. Stationary magnet control experiment. A control experiment, where magnetic particles (MPs) loaded onto the magnetic ratcheting chip dwell 3 cm from N52 nickel plated stationary magnet (2.5 cm x 2.5 cm x 1 cm; surface magnetic flux centered on the axis of magnetization of 0.4933T), demonstrating that on-chip particle movement does not occur using this configuration. The region within the yellow represents the region where the liquid particle suspension was loaded onto the chip.
