# Supplementary material for: Magnetic microparticle concentration and collection using a mechatronic magnetic ratcheting system
Source: PLoS One. 2021 Feb 18;16(2):e0246124. doi: 10.1371/journal.pone.0246124 (PMC7891735; doi:10.1371/journal.pone.0246124)

S2 Fig. Baseline ratcheting experiments. Setting the angle phi (*φ)* at 0° results in differing MP final collection locations. We used 2.8 micro MPs for these experiments. MPs are in the colored fluid and have been outlined in yellow, while MP inlet region is outlined in orange for clarity. A Luer stub (item in red, in the chip collection patch region) was added to facilitate fluid removal from the collection patch region of the chip.


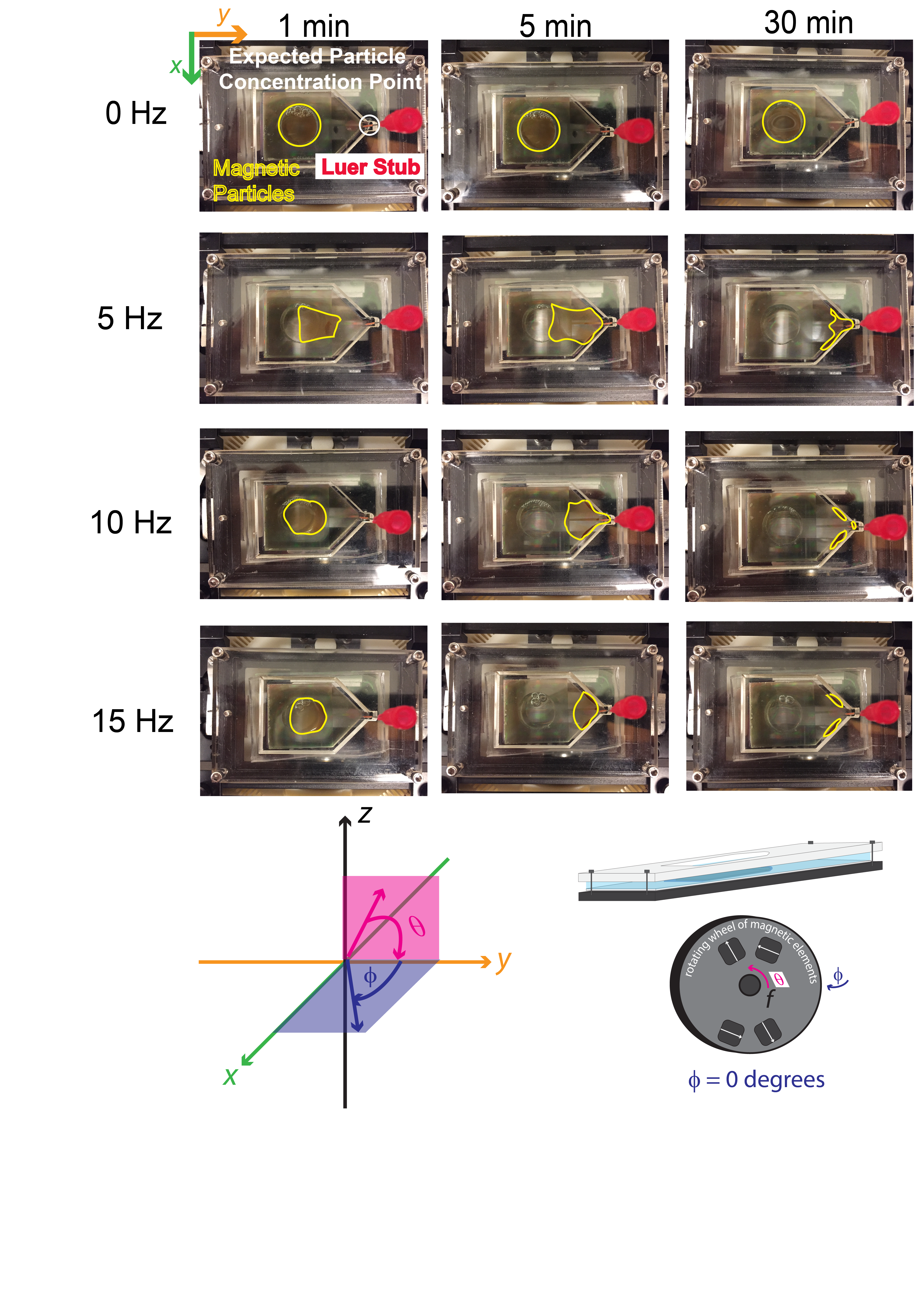

Supplement: S2 Fig — (DOCX) [file pone.0246124.s002.docx]
