# Supplementary material for: Magnetic microparticle concentration and collection using a mechatronic magnetic ratcheting system
Source: PLoS One. 2021 Feb 18;16(2):e0246124. doi: 10.1371/journal.pone.0246124 (PMC7891735; doi:10.1371/journal.pone.0246124)
Supplement: S3 Fig — (DOCX) [file pone.0246124.s003.docx]

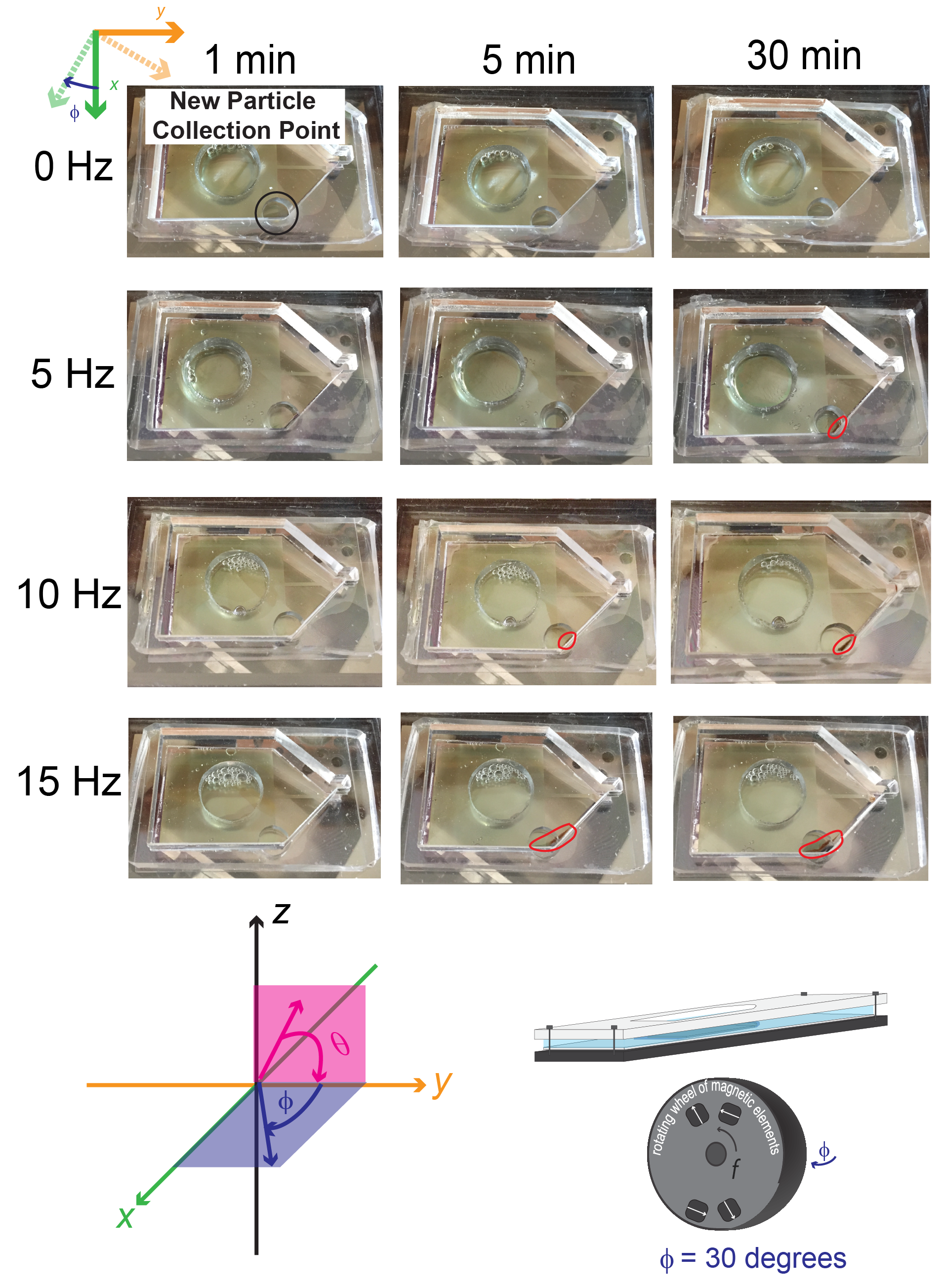


S3 Fig. Ratcheting experiments with adjusted azimuth angle phi (*φ)*. With configuration phi (*φ)* = 30° most MPs concentrate at one discrete location. The 2.8 micro MPs displayed here color the fluid brown and have been outlined in red for clarity.
