# Supplementary material for: Magnetic microparticle concentration and collection using a mechatronic magnetic ratcheting system
Source: PLoS One. 2021 Feb 18;16(2):e0246124. doi: 10.1371/journal.pone.0246124 (PMC7891735; doi:10.1371/journal.pone.0246124)
Supplement: S4 Fig — (DOCX) [file pone.0246124.s004.docx]

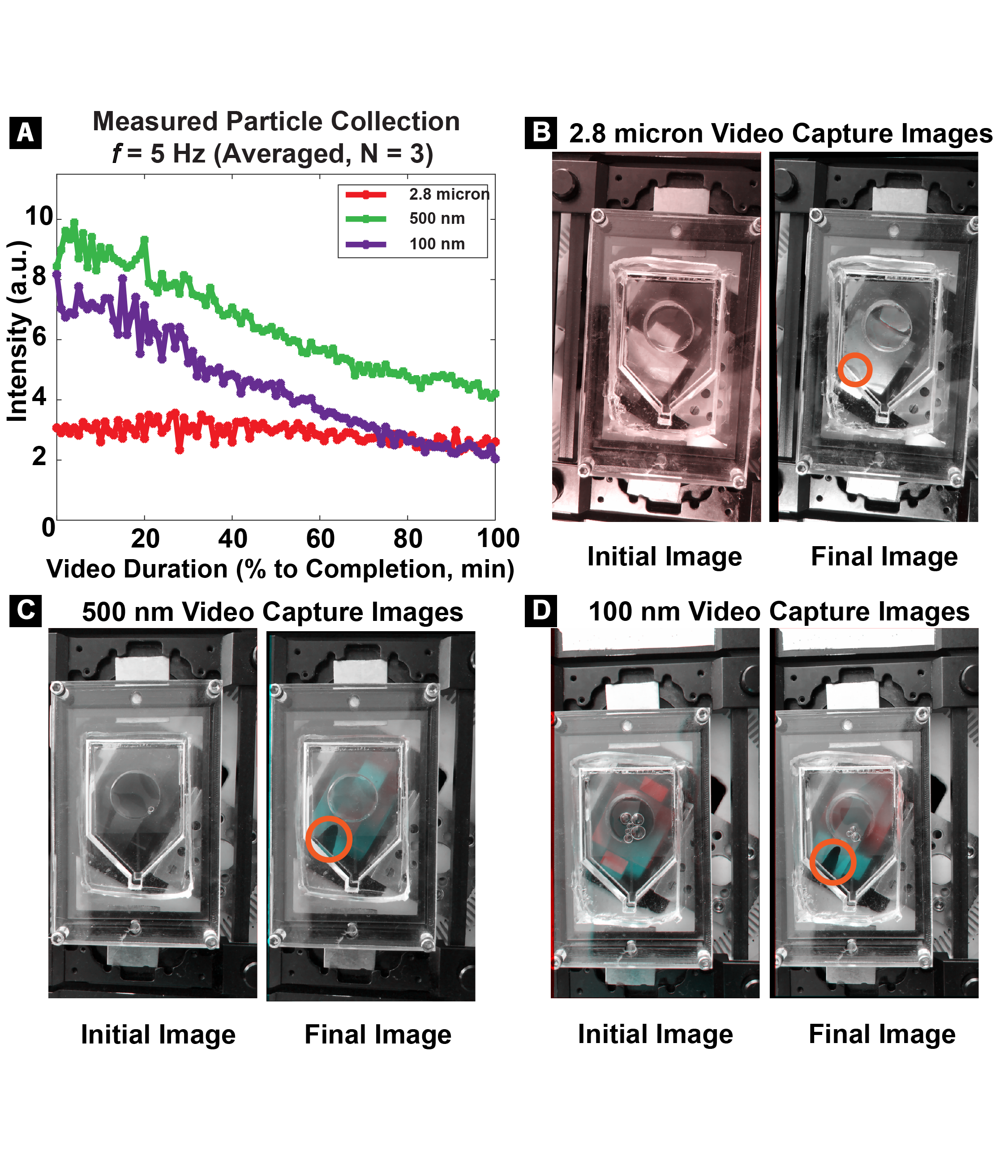


S4 Fig. Ratcheting experiments at frequency (*f*) = 5Hz. A) Ratcheting experiments demonstrating particle accumulation in the collection region, highlighted with a red circle. Ratcheting frequency is 5 Hz. B) 2.8 μm particle video capture still images. C) 500 nm particle video capture still images. D) 100 nm particle video capture still images. For these experiments, the angle phi (*φ)* was set to 30°.
