# Supplementary material for: Magnetic microparticle concentration and collection using a mechatronic magnetic ratcheting system
Source: PLoS One. 2021 Feb 18;16(2):e0246124. doi: 10.1371/journal.pone.0246124 (PMC7891735; doi:10.1371/journal.pone.0246124)
Supplement: S5 Fig — (DOCX) [file pone.0246124.s005.docx]

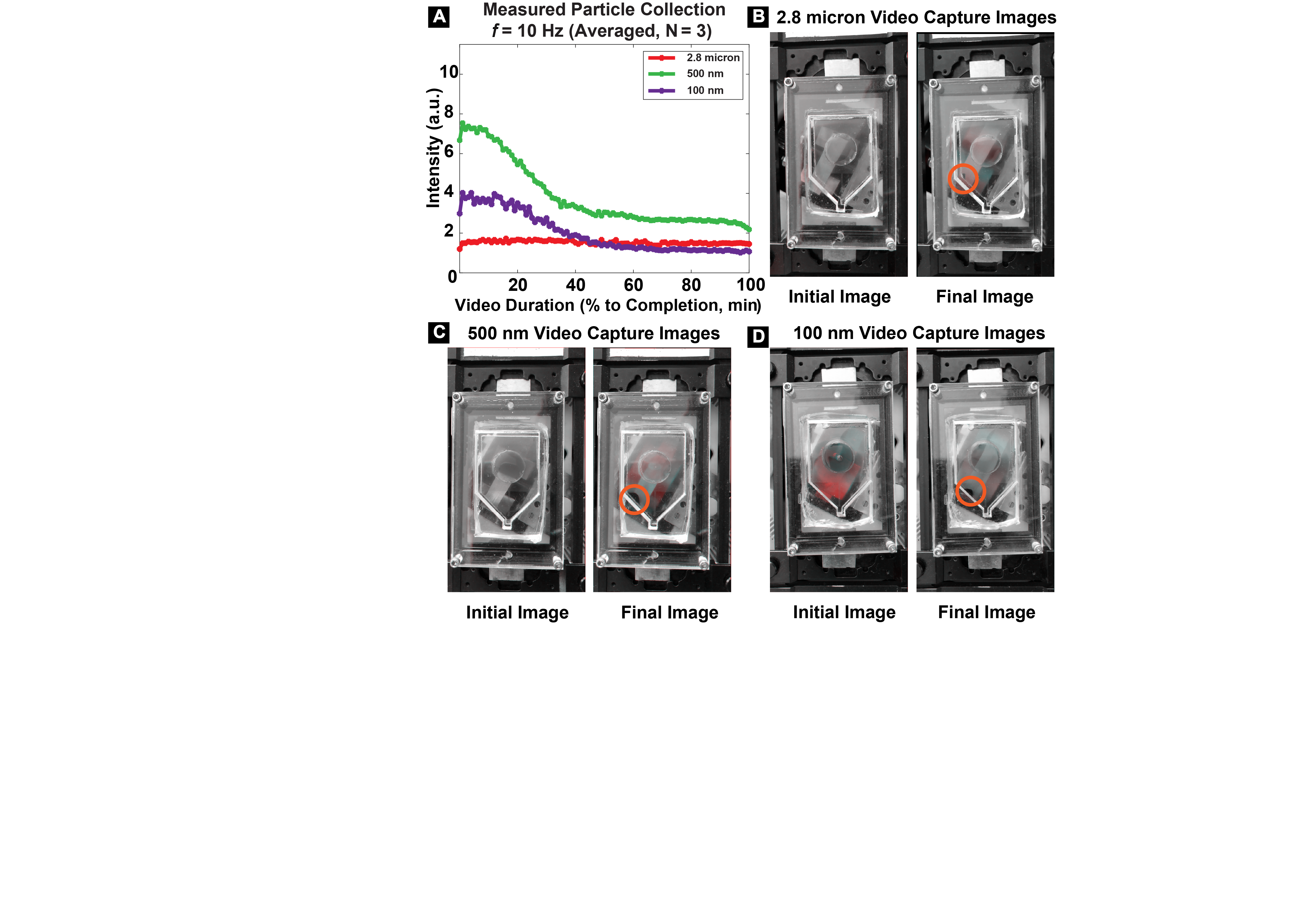


S5 Fig. Ratcheting experiments at frequency (*f*) = 10Hz. A) Ratcheting experiments demonstrating particle accumulation in the collection region, highlighted with a red circle. Ratcheting frequency is 10 Hz. B) 2.8 μm particle video capture still images. C) 500 nm particle video capture still images. D) 100 nm particle video capture still images. For these experiments, the angle phi (*φ)* was set to 30°.
